# Potential relevance of pig gut content transplantation for production and research

**DOI:** 10.1186/s40104-019-0363-4

**Published:** 2019-07-02

**Authors:** Nuria Canibe, Mark O’Dea, Sam Abraham

**Affiliations:** 1 Department of Animal Science, Aarhus University, AU-FOULUM, PO BOX 50, 8830 Tjele, Denmark; 2 Antimicrobial Resistance and Infectious Disease laboratory, College of Science, Health, Engineering and Education, Murdoch University, Western Australia, Australia

**Keywords:** *Clostridium difficile*, Faecal-microbiota-transplantation, Feed conversion, Gastrointestinal tract, Health, Microbiota, Pig model, Pigs

## Abstract

It is becoming increasingly evident that the gastrointestinal microbiota has a significant impact on the overall health and production of the pig. This has led to intensified research on the composition of the gastrointestinal microbiota, factors affecting it, and the impact of the microbiota on health, growth performance, and more recently, behavior of the host. Swine production research has been heavily focused on assessing the effects of feed additives and dietary modifications to alter or take advantage of select characteristics of gastrointestinal microbes to improve health and feed conversion efficiency. Research on faecal microbiota transplantation (FMT) as a possible tool to improve outcomes in pigs through manipulation of the gastrointestinal microbiome is very recent and limited data is available. Results on FMT in humans demonstrating the transfer of phenotypic traits from donors to recipients and the high efficacy of FMT to treat *Clostridium difficile* infections in humans, together with data from pigs relating GI-tract microbiota composition with growth performance has likely played an important role in the interest towards this strategy in pig production. However, several factors can influence the impact of FMT on the recipient, and these need to be identified and optimized before this tool can be applied to pig production.

There are obvious inherent biosecurity and regulatory issues in this strategy, since the donor’s microbiome can never be completely screened for all possible non-desirable microorganisms. However, considering the success observed in humans, it seems worth investigating this strategy for certain applications in pig production. Further, FMT research may lead to the identification of specific bacterial group(s) essential for a particular outcome, resulting in the development of banks of clones which can be used as targeted therapeutics, rather than the broader approach applied in FMT. This review examines the factors associated with the use of FMT, and its potential application to swine production, and includes research on using the pig as model for human medical purposes.

## Introduction

It has long been recognized that the gastrointestinal tract (GI-tract) microbiota of the pig (and mammals in general) has a major impact on the health and development of the host [1–4]. Approximately 10^14^ bacteria inhabit the mammal GI-tract [5–7] and 7,685,872 non-redundant genes have been identified in the pig faecal microbiome [8]. This gives an idea of the complexity of the gut ecosystem, and intuitively, the plethora of possible functions the gut microbiota can have, and the potential influence on the host. In line with this, Isaacson and Kim [9] stated that the genetic diversity of the microbiota in the gastrointestinal tract is immense and has the potential to provide numerous biological activities that the host lacks.

The microbiota profoundly impacts an array of physiological, developmental, nutritional, and immunological processes of the host; and helps protect the animal from colonization or overgrowth of pathogens and other non-desirable species [1, 6, 10–12]. Conversely, the commensal bacteria may have a series of effects that can negatively impact the host, i.e., compete with the host for nutrients, produce toxic compounds, alter intestinal morphology, and induce immune response in the GI-tract, which can impair feed conversion efficiency [11, 13, 14].

Many studies have been conducted dealing with the composition and function of the GI-tract microbiota, the impact of various factors upon it, and the influence of the microbiota on the host. Previously, the microbiota was investigated using predominantly phenotypic methods such as culturing techniques and reporting of metabolite concentrations; and in more recent years, culture-independent molecular techniques, including denaturing gradient gel electrophoresis, terminal restriction fragment length polymorphism, quantitative polymerase chain reaction, 16S rRNA gene amplicon sequencing, and metagenome analysis have been used [1, 15–22]. Further, other techniques, including proteomics, transcriptomics, and metabolomics, have also been used to investigate the function and impact of the GI-tract microbiota on the host [23–27]. In addition, these high resolution techniques are being advanced and combined to examine the genotypic and phenotypic components of the microbiome, in the burgeoning field of integrative omics [28]. All this demonstrates the great efforts being undertaken to decipher the microbial ecosystem of the gastrointestinal tract and its influence on the host.

Richards et al. [11] described very precisely the main objectives of much of the research conducted regarding the gastrointestinal ecology in pigs: i) to determine the optimal microbiota for animal health and performance under commercial growth conditions; and ii) to develop dietary and other interventions to establish this microbiota.

In the search for strategies to improve performance and prevent disease, manipulation of the GI-tract microbiota through different types of feed/feed additives/feeding alternatives have been investigated. These include ingredient composition, organic acids, plant extracts, essential oils, probiotics, prebiotics, feed processing, fermented liquid feed, zinc oxide, copper sulphate, and antimicrobial peptides [1, 16, 29–37]. Only recently has faecal microbiota transplantation (FMT) been investigated for the purpose of GI-tract microbiome manipulation in pigs with the aim of improving phenotypes in these animals.

Faecal microbiota transplantation in pigs, from pig-to-pig or human-to-pig, when used as a model for humans is also a research area of interest and potential [38–46].

Although FMT is an ancient practice, both in humans and animals (see section "A brief history of FMT" for details), FMT in pig production aimed at improving phenotypes in pigs through the establishment of a donor microbiota in a recipient, has only recently been examined [47–52].

The reasons for the recent introduction of this strategy in studies with pigs likely follow various outcomes from human medical research. Studies showing how different phenotypes (obese and lean) in humans could be reproduced in recipient mice by faecal transplantation [53–56], and the use of FMT in humans to treat recurrent *Clostridium difficile* infections (rCDIs) with great success (ca. 90% resolution) [57–62] have opened the door to the possibility of using FMT to treat diseases and alter GI-tract microbiota in pigs.

Furthermore, pig studies reporting evidence that the host gut microbiota is linked to body weight, body weight gain, and feed efficiency [63–68] have promoted the hypothesis that manipulation of the GI-tract microbiota composition/function profile could lead to improved growth traits in pigs.

### Definition

Faecal microbiota transplantation is commonly defined as a strategy to treat disease. The definition proposed by various authors could be summarized as follows: FMT refers to the transplantation of faecal suspension from healthy donors to the GI-tract of a recipient patient, in order to treat a specific disease associated with alteration of gut microbiota, to achieve the treatment of gastrointestinal diseases, to treat dysbiosis-associated disease, to increase intestinal microbial diversity and reestablish a normal microbiome [47, 61, 69–72].

Gupta et al. [73] gave a somewhat different definition, which in principle, does not include a diseased patient: FMT is the administration of a solution of faecal matter from a donor into the intestinal tract of a recipient in order to directly change the recipient’s gut microbial composition and confer a health benefit.

### A brief history of FMT

According to Zhang et al. [74], the first human faecal transplantation dates from the fourth century in China, where the ingestion of a human faecal suspension by patients who had food poisoning or severe diarrhea was described. This gave positive results and was considered a medical miracle. Later, in the sixteenth century, a series of prescriptions using fermented faecal solutions, fresh faecal suspensions, dry feces, or infant feces for effective treatment of abdominal diseases with clinical signs of severe diarrhea, fever, pain, vomiting, and constipation were described [74]. In modern times, the idea of FMT was revived by the work of Eiseman et al. [75], reporting the recovery of four patients with pseudomembranous enterocolitis, which at the time had a 75% mortality rate, after administration of enemas composed of faeces from healthy individuals. *Micrococcus pyogenes*, the agent of the disease, was isolated in the stools of the patients prior to FMT, but could not be detected after treatment.

In the past two decades, FMT has been an emerging field in human medicine. Faecal microbiota transplantation has been established as an effective treatment for rCDIs. The successful use of FMT in managing rCDIs has resulted in the exploration of FMT as a potential treatment for a range of diseases and disorders. This includes inflammatory bowel disease, irritable bowel syndrome, insulin resistance, multiple sclerosis, idiopathic thrombocytopenic purpura, obesity, metabolic disease, and some neuropsychiatric disorders [58, 76–80]. With the exception of the use of FMT for rCDIs and inflammatory bowel disease, the studies in other diseases and disorders are small and not repeated in sufficient numbers to allow solid conclusions to be drawn.

In veterinary medicine, the first reports on transplantation of viable enteric bacteria, denoted ‘transfaunation’, are considered to be those by the Italian anatomist Fabricius Aquapendente in the seventeenth century. He observed that cud taken directly from a healthy ruminant and placed in the mouth of an animal that had lost its capacity to ruminate would result in restoration of rumination and health [81]. DePeters and George [82] described the earliest printed reference about transfaunation in Sweden to be from 1776 (Hjortberg) that stated “It is common practice, even in the country side, to take the fodder out of the mouth of a sheep or a goat to give it to an animal which does not ruminate”. Brag and Hansen [83] also reported that the Swedish peasants used to administer living ruminal microorganisms from a healthy cow or sheep to an animal suffering from ruminal indigestion by giving the diseased animal a cud bolus obtained from the healthy animal. DePeters and George [82], in their review, concluded that rumen transfaunation is a widely accepted, successful procedure to treat simple indigestion in ruminants. Further, the procedure has also clinical applications for post-operative treatment of cattle with left sided abomasal displacements [82].

Mullen et al. [84] in their review stated that whilst there are no peer-reviewed studies of FMT in horses, equine practitioners have a history of providing nasogastric administration of ‘faecal tea’ from healthy horses to horses with diarrhoea with anecdotal reports of success.

Faecal microbiota transplantation has also been investigated in poultry. For example, Nurmi and Rantala [85], in a challenge study, observed a reduced susceptibility to *S. infantis* infection in chicks administered with digesta from healthy adult cocks. More recently, other studies have aimed to improve parameters such as feed efficiency in chickens using the FMT technique [86].

Regarding pigs, FMT has only recently been investigated as a strategy to improve phenotypes with regards to health and feed efficiency [47–52, 87, 88] (Canibe et al., unpublished). On the other hand, there is a longer history in the use of FMT and pigs when transplanting human faeces to pigs with the aim of obtaining human microbiota-associated (HMA)-pigs to be used as a model for humans [38–42, 89].

The previous illustrates that the practice of FMT is ancient, practiced long before the current understanding of gut microbiome and its influence on the host, and has recently gained interest in several areas including medicine, nutrition, and immunology, both in humans and animals. As Aroniadis and Brandt [58] pointed out, FMT received public attention more recently after several studies were published showing that stool is a biologically active, complex mixture of living organisms with great therapeutic potential for CDI and perhaps other GI-tract and non-GI-tract disorders. Therefore, FMT in pig production is investigated in the context of developing effective alternative feeding strategies and production practices to improve performance or reduce the use of antibiotic and heavy metals in order to alleviate problems like bacteria resistance and environmental concerns, without impairing animal welfare and growth performance.

### Faecal microbiota transplantation in humans

#### Faecal microbiota transplantation and *Clostridium difficile* infection

*Clostridium difficile* infections are a leading cause of diarrhoeal disease in health care and community settings, associated with severe morbidity and mortality world-wide [90]. Clinical manifestation of CDIs ranges from mild to moderate diarrhoea to toxic megacolon, colonic perforation, and death [90]. Globally, since the early 2000s, there has been an increase in incidence, severity, and mortality of CDIs. This is largely attributed to the emergence of previously rare, epidemic fluoroquinolone-resistant strains associated with increased virulence [90, 91]. The key factor influencing the occurrence of CDIs is exposure to antimicrobials followed by the disruption of normal colonic microbiota. This results in the depletion of normal colonic microbiota, which facilitates the proliferation of endogenous or environmental *C. difficile* to proliferate in the colon and produce toxins [91].

Since the hallmark of CDIs is the alteration of colonic microbiota, restoration of this microbiota via FMT has been utilized for the treatment of recurrent or relapsing CDIs [92–94] and moderate CDIs that fail to respond to standard antimicrobial therapy [57]. The rationale behind the approach is to re-establish the dynamics and diversity of the microbiota, resulting in a return to normal function of the colonic microbiota.

Faecal microbiota transplantation has been offered in select centers across the world for decades, primarily as a last effort to treat rCDI, which is characterized by rapid infection recurrence upon antibiotic discontinuation. Faecal microbiota transplantation has shown to be highly effective in rCDI infection with about 85–90% of patients being cured after FMT treatment [57, 58, 60–62, 73]. The effectiveness of FMT on refractory CDI (when patients do not respond to the antibiotic treatment) is less solidly established than on rCDI. Although a few studies have reported high resolution rates [95–97], in general, lower efficacy has been observed [60, 61, 92]. Also, there is insufficient evidence to recommend FMT as a treatment for the first episode of CDI [61]. Consequently, the Food and Drug Administration in the USA has approved the use of FMT as an investigational drug for the treatment of rCDIs after failure of standard antimicrobial use [94] and the European Society for Microbiology and Infectious Disease recommends FMT as a treatment for rCDI [61, 98].

#### Administration method/route

A number of administration routes have been explored for the FMT treatment of CDIs. Administration of fresh or frozen homogenized faecal suspensions using nasogastric/nasoduodenal/nasojejunal tubes, gastroscopy, rectal tube/enema, and colonoscopy has been utilized. A review by Postigo and Kim [99] compared colonoscopy and nasogastric tube for the administration of FMT. Nasogastric tube insertion does not require endoscopy guidance or bowel preparation, with the advantage of greater accessibility and ease of use. On the other hand, colonoscopy may have better therapeutic potential than any other modalities by having the capacity to deliver fecal infusion directly to the colon. Both routes appeared to be highly effective. In a small study involving 20 patients, oral frozen encapsulated inoculum from unrelated donors has also been used for the treatment of rCDI with significant success rates (90% resolution of diarrhea) [100]. According to Cammarota et al. [61], many systematic reviews and meta-analyses have reported that colonoscopy achieves higher resolution rates of rCDI and similar safety profile than other routes of delivery.

#### Donor characteristics

Choice of donors for the FMT treatment can vary, ranging from family members, intimate partners, house mates, and volunteer donors [101]. Although there have been no adverse safety issues with FMT treatment, donor screening is essential to minimize the risk of transmission of communicable diseases. In addition, a comprehensive risk assessment of the donor is required to estimate the risk of a recent contraction of infectious disease and rule out potential exposure to other infectious agents that are not identified by currently available laboratory methods [101].

#### Recipient preparation

One of the key considerations for recipient preparation is the cessation of antimicrobial treatment 1–3 days prior to FMT. When rectal tube/enema or colonoscopy is used, bowel lavage prior to FMT administration on the recipient is common to flush residual faeces, antimicrobial residues, and *C. difficile* bacteria, spores and toxins; and/or anti-diarrhoeals to prolong retention of the faecal suspension in the colon [57, 61, 101, 102]. When the upper route is used, proton pump inhibitors are administered, although their beneficial effect has not been proven [61].

### Faecal microbiota transplantation in mouse models

Faecal microbiota transplantation studies in mice have shown promising results in a number of areas including obesity, reversion of the dysbiotic effects of antimicrobial use and chemotherapy, improved disease resistance, and enhancing immune function. Evidence from mechanistic studies suggest that obesity and associated metabolic disorders are linked to composition and function of the gut microbiota of the host [103]. Using mouse models, a number of studies have investigated the role of gut microbiota and FMT in controlling body weight and obesity. Studies in mice have shown that diet shapes the gut microbiota [104] and microbiota from obese individuals have enhanced ability to harvest energy from diet and energy stores [53, 105]. Using germ free mice, researchers have demonstrated that obese traits attributed to the microbiota are transmissible via FMT [55, 106]. Turnbaugh et al. [53] illustrated that germ free mice receiving microbiota from obese mice developed increased body fat compared to those receiving microbiota from lean mice. Another mouse experimental trial demonstrated that FMT may assist in preventing or reversing acute intestinal inflammation and mucosal barrier function following the administration of antimicrobials and chemotherapy [107].

A study by Rosshart et al. [108] also showed that gut microbiota of wild mouse can be viably preserved and successfully transferred to laboratory mice, and the newly transferred microbiota can be maintainable over several subsequent generations of the recipient mice. The study also showed that gut microbiota of wild mice promoted host fitness and improved resistance to infectious diseases such as influenza A and mutagen- and inflammation-induced colorectal tumorigenesis [108].

In recent years, efforts have also been made to use mouse models that resemble human microbiota to study various human diseases and host-microbe interaction. A number of experimental studies have successfully engrafted core human microbiota in both germ free and mice treated with antimicrobials (HMA-mice) [55, 56, 109]. A comprehensive study by Riduara et al. [56] demonstrated that FMT from adult female human twin pairs discordant for obesity into germ-free mice resulted in successful and reproducible transmission of donor body composition phenotype [56]. A similar phenomenon was also observed in germ free mice by inoculating cultured anaerobic bacterial collection from human twin pairs. In addition, this study was also successful in transplantation of human microbiota in germ free mice with the preservation of the taxonomic and functional features of the donor microbiota. It should be noted that not all aspects of human microbiota and associated functions are preserved in these mouse models.

### Faecal microbiota transplantation in pig production

The aforementioned work in humans and mice are encouraging with regards to the use of FMT to promote outcomes like feed efficiency, disease prevention and treatment. However, the translation of this research into practical applications for pigs require well designed and executed, randomized control trials.

There are few studies in which FMT in pigs has been investigated with the focus on pig production, rather than as a model for human research, and they have all been recently published [47–52, 72, 87, 88]. Besides these, some other studies, although not using the pig as a model for humans, have used FMT in trials with gnotobiotic or cesarian-section delivered pigs investigating the impact of being colonized by a simple versus a complex microbiota (FMT) e.g., [2, 110, 111]. These studies show an impact of colonization on microbiota composition and gut maturation in the recipients. However, this aspect of investigating the role of the gut microbiota by comparing conventional animals with those without a GI-tract microbiota or a simple one on various parameters of the pig physiology and metabolism is beyond the purpose of this review.

Some studies have examined various parameters related to intestinal health, including intestinal development, the epithelial barrier, and microbiota composition [47–51, 88]. Others have investigated the use of FMT as a possible strategy to improve feed efficiency of recipient pigs [52], Canibe et al. (unpublished); and others have tested the potential of FMT to prevent or reduce disease [48, 72].

#### Description of studies

The experimental design of recently published studies (Table 1) differ significantly depending on the hypothesis being investigated, and to this end it is difficult to draw a clear conclusion on the combined results. Below we discuss the findings of the studies separately, and how these relate to improved gut function in the pig.

**Table 1:** Experimental design of studies on faecal microbiota transplantation in pig production

Ref^a^	Hypothesis/Aim	Treatment	No. of pigs/group	Start age, d	Duration, d	Transplantation method	Conclusions
1	Effect on gut bacterial community structure, gut barrier and growth performance	Control (saline)	6 litters; 9–10 pigs/litter	1	27	1.5 mL daily, from d 1 to d 11. Orally	The recipients’ resistance to disease was enhanced, diarrhea was reduced and weight gain was raised
		Faeces from Jinhua adult pigs	6 litters; 9–10 pigs/litter			1.5 mL daily from d 1 to d 11. Orally	
2	FMT regulates intestinal mucosal autophagy and anti-inflammatory ability	Control (phosphate-buffered saline)	6	1	14	1.5 mL daily, from d 1 to d 11. Orally	FMT triggered intestinal mucosal protective autophagy
		Faeces from Jinhua adult pigs	6			1.5 mL daily from d 1 to d 11. Orally	
3	Changes in the gut microbiota induced by FMT alter its metabolic function, which might regulate mucosal integrity and immune responses	Control (PBS)	6	1	14	1.5 mL every second day, from d 1 to d 14. Orally	FMT reduced susceptibility to LPS-induced destruction of epithelial integrity and severe inflammatory response
		Control (PBS) (+ NaCl at slaughter)	6			1.5 mL every second day, from d 1 to d 14. Orally	
		Control (PBS) (+ LPS at slaughter)	6			1.5 mL every second day, from d 1 to d 14. Orally	
		Faeces from Jinhua adult pigs	6			1.5 mL every second day, from d 1 to d 14. Orally	
		Faeces from Jinhua adult pigs (+ LPS at slaughter)	6			1.5 mL every second day, from d 1 to d 14. Orally	
4	Effects of early fecal microbiota transplantation on gut development in sucking piglets	Control (saline)	6	3	56	10 mL daily from d 1 to d 3; 10 mL every second day from d 4 to d 15; 20 mL every 5 days from d 16 to d 46. Intragastrically	FMT from the Yorkshire and Rongchang pigs to DLY piglets damaged the gut microbiota balance and thereby intestinal health
		Faeces from 5 Tibetan pigs (12 weeks of age)	6			10 mL daily from d 1 to d 3; 10 mL every second day from d 4 to d 15; 20 mL every 5 days from d 16 to d 46. Intragastrically	
		Faeces from 5 Yorkshire pigs (12 weeks of age)	6			10 mL daily from d 1 to d 3; 10 mL every second day from d 4 to d 15; 20 mL every 5 days from d 16 to d 46. Intragastrically	
		Faeces from 5 Rongchang pigs (12 weeks of age)	6			10 mL daily from d 1 to d 3; 10 mL every second day from d 4 to d 15; 20 mL every 5 days from d 16 to d 46. Intragastrically	
5	To identify and validate gut microbes associated with diarrhoea resistance	Control (none)	3	1 post-weaning	11	None	FMT reduced diarrhoea in recipients; FMT caused shifts in the microbiota of recipients towards that of donors
		Control (saline)	3			2 mL every second day from d 10 to d 18. Orally	
		Faeces from Congjiang weaners (high dose)	3			2 mL every second day from d 10 to d 18. Orally	
		Faeces from Congjiang weaners (low dose)	3			2 mL every second day from d 10 to d 18. Orally	
		Oxytetracycline	3			2 mL at weaning. Intramuscular	
6	The gastrointestinal microbiome could be strengthened or weakened by feeding maternal fecal microbiota or antibiotics	Control (saline)	5	1	21	3 mL every day from d 1 to d 6. Orally	FMT showed beneficial effects on GI-tract microbiota and the metabolic profiles of piglets on day 7, while less effect on day 21
		Amoxicillin	3			3 mL every day from d 1 to d 6. Orally	
		Faeces from the dam	5			3 mL every day from d 1 to d 6. Orally	
7	Whether FMT in sows and/or neonatal offspring with inocula from highly feed-efficient pigs could improve offspring feed efficiency	Control (saline)	18/12	d 70 of gestation	185	None	Reduced body weight, poorer absorptive capacity and intestinal health after FMT
		Faeces from 4 finisher pigs with the lowest RFI to piglets at birth	18/12			8 mL at birth. Orally	
		Faeces from 4 finisher pigs with the lowest RFI to piglets at birth, 3, 7, and 28 d of age	18/12			8 mL at birth, 3, 7, and 28 days of age. Orally	
		Faeces from 4 finisher pigs with the lowest RFI to sows	18/12			200 mL d 70 and d 100 of gestation. Intragastrically	
		Faeces from 4 finisher pigs with the lowest RFI to sows, and piglets at birth	18/12			200 mL d 70 and d 100 of gestation. Intragastrically. 8 mL at birth to piglets. Orally	
		Faeces from 4 finisher pigs with the lowest RFI to sows, and piglets at birth, 3, 7, and 28 days of age	18/12			200 mL d 70 and d 100 of gestation. Intragastrically. 8 mL at birth, 3, 7, and 28 days of age. Orally	
8	Microbiota from donors differing in composition can be established in recipient pigs	Colon digesta from growers (18 weeks old) fed a Control diet	20	28	88	20 mL on d 4 and d 18 post-weaning. Orally	Microbiota did not established in the recipients
		Colon digesta from growers (18 weeks old) fed a Control diet added 170 ppm copper	20			20 mL on d 4 and d 18 post-weaning. Orally	
		Colon digesta from growers (18 weeks old) fed a Control diet added 40 ppm tylosin	20			20 mL on d 4 and d 18 post-weaning. Orally	
		Colon digesta from growers (18 weeks old) fed a Control diet added 1% benzoic acid	20			20 mL on d 4 and d 18 post-weaning. Orally	
9	To assess the alleviation of epithelial injury in the *Escherichia coli* K88-infected piglets following FMT	Control	6	1	21	None	Epithelial injury was alleviated in the *E. coli* K88-infected piglets following FMT
		Challenged *E.coli* K88 + PBS	6			100 mL K88 from d 15 to d 17+ 100 mL PBS on d 18 to d 20	
		Challenged *E. coli* K88 + faeces	6			100 mL K88 from d 15 to d 17+ 100 mL faeces on d 18 to d 20	
10	FMT prior to co-infection with PRRSV and PCV-2 reduces clinical signs and pathology associated with PCVAD	Control (saline) + (PRRSV+PCV-2d)	10 (in 1 pen)	25	51	5 mL every day from d 1 to d 7 post-weaning	FMT decreases the severity of clinical signs following co-infection with PRRSV and PCV-2 by reducing the prevalence of PCVAD
		Faeces from 2 sows (+PRRSV+PCV-2d)	10 (in 1 pen)			5 mL every day from d 1 to d 7 post-weaning	

^a^1: Hu et al. [47]; 2: Cheng et al. [48]; 3:Geng et al. [50]; 4: Diao et al. [49]; 5: Hu et al. [51]; 6: Lin et al. [88]; 7: McCormack et al. [52]; 8: Canibe et al. (unpublished); 9: Cheng et al. [48]; 10: Niederwerder et al. [72]

Hu et al. [47] conducted a study in which faeces from Jinhua pigs, more resistant to challenge by enterotoxigenic *Escherichia (E.) coli* K88 [112], were transplanted to DLY (Duroc × Landrace × Yorkshire) newborn piglets. The authors hypothesized that FMT would modulate the gut microbiota composition, and improve intestinal barrier and immune function in the piglets. They observed a higher average daily body weight gain and lower diarrhea incidence in the piglets receiving FMT compared to the control group during the first four weeks after birth. Faecal microbiota transplantation had no impact on the richness or α-diversity of the microbial community. The relative abundance of Firmicutes in the colon of recipient piglets was higher, and that of Proteobacteria lower compared to the Control group. At the genus level, *Prevotella*, *Oscillospira*, *CF231*, and *Ruminococcus* were more abundant, whereas *Bacteroides*, *j2–29*, *Sutterella*, and *Escherichia* were less abundant in the recipient piglets at one or both sampling times.

The impact of FMT on various parameters related to epithelial barrier functions included up-regulation of mRNA and protein expressions of the gel-forming mucin 2 (MUC2), and relative expression of the tight junction proteins zonula occludens-1 (ZO-1) and occludin (OCLN) in ileum and colon in recipient piglets. In addition, the number of goblet cells was higher in the ileum and colon of the FMT piglets, which could explain the higher expression of MUC2. These results taken together were considered as indicators of a beneficial effect of the FMT on the development of the intestinal mucous barrier in recipient piglets. In accordance, scanning electron microscope images of the villi in jejunum of recipient piglets indicated improved morphology as compared to the donors.

The relative protein expression of ß-defensin 2 in the ileal mucosa was increased in the recipient piglets, as well as the relative expression of Toll-like receptor 2 and Toll-like receptor 4, and the optical density of secretory IgA cells in the colon. Increased expression of ß-defensin 2, an antimicrobial peptide with a higher expression level in the intestine of Jinhua pigs than in Landrace pigs [47], was interpreted as playing a key role in contributing to the improvement of the resistance of recipients to disease. The results of this study indicated that FMT changed the population structure of intestinal microbiota, which contributed to the improvement of intestinal morphology, the development of the intestinal mucosal barrier, and innate immunity in recipient piglets.

The same authors, Cheng et al. [48], conducted another study with a similar transplantation procedure to that used in their previous work [47]. By performing proteomic analysis of colonic mucosal samples, differentially expressed proteins between the donors and recipients included proteins involved in multiple processes, such as energy production, lipid and amino acid metabolism, autophagy, oxidative stress, and inflammatory responses. The focus of the study was the impact of FMT on mucosal autophagy, which has been reported as essential in host defense against invasive bacteria [113]. The levels of autophagy-related proteins in the forkhead box O signaling pathway and the antioxidant protein superoxide dismutase 2 were increased, whereas the levels of proteins related to inflammation response, were decreased in the recipient. Therefore, the results suggested that FMT triggered mucosal protective autophagy and thereby protected the integrity of the intestinal barrier.

In another study, Geng et al. [50] investigated FMT as a strategy to maintain intestinal homeostasis by regulating mucosal integrity and immune responses in piglets. Performing FMT to lipopolysaccharide (LPS)-treated piglets significantly alleviated the decrease in colonic crypt depth/tissue thickness ratio induced by LPS challenge; increased the height and quantity of microvilli as well as the distribution of epithelial cell junctions in colon; prevented the LPS-induced reduction in epithelial Ki67-positive cells (a measure of cell proliferation); increased the relative mRNA expression of adherens junction protein E-cadherin (provides cell-cell adhesion in relation to epithelial barrier); and increased the relative mRNA expression of the anti-inflammatory cytokine transforming growth factor-β1, while reduced that of the pro-inflammatory cytokines interleukin (IL)-1β, IL-6, tumour necrosis factor-α, and interferon-γ, and that of chemokine monocyte chemotactic protein 1. These results suggested that FMT can help to alleviate the disruption of the epithelial barrier and the inflammatory responses caused by LPS challenge.

Regarding microbiota composition in colon digesta, no effect of FMT on diversity at operational taxonomic unit (OTU) level was detected. Alpha-diversity indices were significantly higher at the class and order level in the FMT group as compared to the control group; and significant differences in the composition of gut microbiota between the two groups was detected at phylum, family, and genus level, arguably indicating an overall healthier microbiome profile. The metabolome profile of colon digesta of the control group and the FMT group clustered separately. Further, metabolite set enrichment analysis showed that tryptophan metabolism was the most significantly affected metabolic pathway in the recipient colon, which was interpreted as playing a role in maintenance of the intestinal barrier following FMT.

The impact of transplanting faeces with varying bacterial composition was investigated by Diao et al. [49] by using donor pigs of three different breeds (Tibetan, Yorkshire, and Rongchang) to suckling (DLY) piglets.

Piglets receiving faeces from Yorkshire and Rongchang pigs had a higher post-weaning diarrhea index than control piglets receiving saline and those receiving faeces from Tibetan pigs. In comparison to the control, the group transplanted with Yorkshire faeces showed various negative responses related to epithelial barrier, i.e., lower mRNA expression of the tight junction protein ZO-1 in ileum, lower goblet cell numbers in ileum and colon, and lower mucin 1 (MUC1) expression in colon; intestinal development, i.e., lower expression of glucagon-like peptide-2 (GLP-2), angiogenin 4 (ANG-4), and insulin like growth factor-1 receptor (IGF-1R) in ileum; digestion and absorption, i.e., lower lactase and γ-glutamyltransferase (γ-GT) activity in the jejunum, lower expression of zinc transporters-1 (ZNT-1) in duodenum and jejunum, of divalent metal transporter-1 (DMT1) in jejunum, and of solute carrier family 7 (SLC_7_A_1_) in ileum; and gut health, i.e., high serum concentration of LPS, and lower superoxide dismutase activity (SOD) in jejunum. Similarly, the piglets receiving faeces from the Rongchang pigs showed various negative responses compared to the control group: lower villous height in ileum; lower expression of GLP-2 in ileum, ANG-4, and IGF-1 in colon; lower lactase and γ-GT activity in jejunum; lower total tract digestibility of dry matter, crude protein, energy, crude ash, ether extract, and calcium; lower expression of regeneration protein IIIγ in colon; lower expression of ZNT-1 in duodenum, and DMT1 in jejunum; and lower SOD activity in jejunum.

On the other hand, the impact of transplantation of faeces from Tibetan pigs was smaller, and considered more beneficial: higher level of the digestive and absorptive enzyme activities Na^+^, K^+^-ATPase, and Ca^+^, Mg^+^-ATPase enzymes in jejunum; higher expression of SLC_7_A_1_ in duodenum; higher expression of the anti-inflammatory cytokine IL-10 in colon; and higher numbers of lactobacilli in cecum and colon compared to control pigs.

The data indicated that the impact of transplanting faeces from the different donor breeds [49] was different, that is, faecal microbiota from Yorkshire and Rongchang pigs to DLY suckling piglets had adverse effects on gut development and function, whereas transplantation of faecal microbiota derived from the Tibetan pigs had a lower impact on the recipients, with some positive effects on intestinal health and function.

Hu et al. [51] conducted a series of studies to investigate the mechanism behind the positive effects observed by FMT and which gut microbes confer this efficacy. This was done in the context of finding alternatives to antibiotics to prevent diarrhea in early-weaned piglets. Faecal microbiota from healthy Congjiang miniature piglets, which were considered to have stronger resistance to post-weaning diarrhoea than Landrace × Yorkshire (LY) piglets, was administered to LY piglet recipients orally prior to weaning. Transplantation at a low dose (and not at a high dose) significantly decreased diarrhea in recipient piglets.

Faecal transplantation affected both α- and ß-diversity and the functional profile of the microbiota. Five bacterial species (*Lactobacillus frumenti*, *L. gasseri* LA39, *Butyricicoccus pullicaecorum*, *Eubacterium hallii*, and *Blautia hansenii*) exhibited significantly higher relative abundance in the FMT piglets than in the Control group at all sampling times. A follow up study with piglets in which the five-bacteria consortium or each bacterium separately were transferred orally showed that the consortium and *L. gasseri* LA39 or *L. frumenti* alone significantly decreased diarrhea. Further studies indicated that the bacteriocin gassericin A, produced by *L. gasseri* LA39 and *L. frumenti*, conferred diarrhea resistance by increasing intestinal fluid absorption and decreasing intestinal fluid secretion.

Thereby, the data suggested that *L. gasseri* LA39 and *L. frumenti* may be worth testing further as candidate bacteria for preventing diarrhea in piglets. Another finding in this study was that the anti-diarrhoea effects of FMT were stronger using a low-dose faecal suspension than upon using a high-dose faecal suspension, suggesting that the impact of FMT may be dose dependent.

Lin et al. [88] used FMT to suckling piglets with maternal faeces as a strategy to positively affect the process of gut microbiota colonization in piglets. Differences in abundance of some bacterial members in stomach, ileum and colon digesta, as well as of the metabolic profile in colon digesta were observed between the Control and FMT group. However, the impact of these changes on the health of the recipients was not clear.

One of the studies investigating FMT as a strategy to improve feed efficiency in recipients by using faeces from highly feed-efficient donors is that of McCormack et al. [52]. In their study, faeces from finisher pigs with low residual feed intake (high feed efficiency) were used and transplanted either to sows and or to their offspring, i.e., the impact of FMT given to sows, or the offspring, or to both was investigated.

A negative impact of FMT on performance was measured, since offspring from transplanted sows were lighter than offspring from control sows, and transplanted offspring were also lighter than control offspring. Numerous differences in microbiota composition were detected as a result of FMT at the phylum and genus levels and at the various sampling time points and sites. Some of the changes in microbiota composition were discussed by the authors as possible contributors to the impaired growth of the offspring due to FMT, including a higher abundance of Bacteroidetes, which have been linked with lower adiposity in pigs; increased abundance of *Bacteroides* and of *Prevotella*, related to lower adiposity in pigs and poorer feed efficiency, respectively; and reduction in *Faecalibacterium*, known for its anti-inflammatory properties and linked with heavier body weight in pigs. Further, FMT practiced to sows or to the piglets impacted predicted functions of the microbiota, which belonged to mainly carbohydrate and amino acid and lipid metabolism and were mostly in the ileum.

The offspring from transplanted sows compared to those from control sows showed various characteristics related to epithelial barrier and possibly absorptive capacity, probably caused by changes in microbiota composition, which were hypothesized by the authors to explain the negative impact of FMT on performance of the recipients. These included lower number of duodenal goblet cells but higher number of ileal goblet cells per μm of villus height; reduced jejunal villus height-to-crypt depth ratio, reduced ileal villus height, width, and area, and lower ileal crypt depth; and upregulation of the genes encoding the tight junction proteins ZO1 and OCLN. Upregulation of the gene encoding the tight junction protein OCLN was also detected in the offspring subjected to FMT. Interestingly, in contrast to [47] and others, e.g. Ulluwishewa et al.[114] and Robinson et al. [115], who considered the tight junction protein expression levels to be associated with improved intestinal barrier integrity, McCormack et al. [52] interpreted the upregulation of *OCLN* and *ZO1* genes as a factor contributing to impaired absorptive capacity, due to a more selective duodenal paracellular permeability. Further, McCormack et al. [52] also considered that the greater number of goblet cells might have resulted in overproduction of mucin in the ileum, forming a physical barrier that decreased nutrient absorption. These responses together with the reductions in ileal villus height and area observed would lead to an impaired nutrient absorption in FMT piglets.

Furthermore, multiple inoculations in offspring amplified the negative impact in some instances. Also, the combinative effect of maternal and offspring FMT showed additive negative effects seen as a much lower slaughter weight and a greater impact on ileal villus height in pigs on the combined treatments than in FMT-treated offspring from control sows.

In line with the objective of the study of [52], Canibe et al. (unpublished) conducted a study to investigate whether transplanting the colonic microbiota from pigs fed diets known to result in high feed efficiency would establish in recipient pigs. The aim being to achieve an improved feed efficiency in the recipient pigs. The experimental diets fed to the donors were a control diet, and control diet with the addition of 170 ppm copper as copper sulphate, 40 ppm tylosin, or 1% benzoic acid. The preliminary results from this study indicated that FMT had only a small impact on the microbiota composition of the recipient pigs as measured 10 weeks after the last transplantation. In order to get more in-depth information on the potential impact of FMT on the host’s metabolism, samples will be further explored by deep metagenomics sequencing.

A few studies have investigated FMT as a strategy to ameliorate the negative impact of bacterial or viral infections in pigs.

Cheng et al. [48] investigated whether FMT could reduce the negative impact of *Escherichia* *coli* K88 infection on the gastrointestinal epithelium of piglets, and positive results were reported. Faecal transplantation alleviated the negative impact the *E. coli* K88 infection had on weight gain and diarrhea incidence on the piglets. Further, the damage caused by *E. coli* infection on jejunal villi was alleviated by FMT; the number of goblet cells and the protein levels of MUC2, ZO-1, and OCLN in colonic mucosa of the infected piglets receiving FMT was higher; and the serum diamine oxidase activity and D-lactate (also used as indicators of intestinal barrier) lower as compared to the infected piglets not receiving FMT.

Niederwerder et al. [72] conducted a study to test the potential of FMT to prevent porcine circovirus associated disease (PCVAD) in a challenge study with pigs co-infected with porcine circovirus type 2 (PCV-2) and porcine reproductive and respiratory syndrome virus (PRRSV). According to Niederwerder et al. [72], in co-infection studies, the presence of increased microbiome diversity is linked to a reduction in clinical signs, an outcome that can be hypothesized to be manipulated by providing FMT.

The FMT treated group had lower morbidity, lower mortality, and fewer pigs showed weight loss due to PCVAD. In general, virus replication during peak clinical disease was reduced in the FMT group; and the production of antibodies directed at both PRRSV and PCV-2 was higher and at more sustained levels. No impact of FMT on bacterial diversity or global changes in bacterial composition was detected. Some differences between the groups were found, though, the FMT having a higher relative abundance of Veillonellaceae, Lachnospiraceae, and Ruminococcaceae in faeces. These changes were hypothesized to partly explain the beneficial impact of FMT by helping the host hydrolyzing feed substrates. Modulation of the systemic immune response by FMT was also speculated to contribute to the results. However, the number of pigs used in this study was low, and it is unclear if PCVAD or PRRSV-associated disease was the underlying cause of clinical disease, due to the sporadic, multifactorial nature of PCVAD.

#### Discussion of the results

The studies presented above and summarised in Table 1 display both positive and negative results [47–52, 72] showing that the possibility to reprogram the porcine intestinal microbiota via FMT, with resultant alterations in host phenotype exists, although the mechanisms and optimum protocols are not clear.

Many factors can contribute to the varying outputs and given the importance of this aspect, there have been attempts to standardize procedures to prepare FMT [116]. The study of Diao et al. [49] suggests that donor is an important factor, since, with all other factors similar, the response of the recipients differed depending on the donor microbiota. Further, the results of Hu et al. [51] showing that the anti-diarrhoea effects of FMT were stronger using a low-dose faecal suspension than upon using a high-dose faecal suspension, and those of McCormack et al. [52] showing that multiple FMT inoculations in offspring amplified the negative impact in some instances could suggest that higher doses of transplant microbiota are not necessarily better, perhaps because some remnant microbiota needs to be present in the recipient or the level of ‘tolerance’ to bacteria from another subject has a limit. Characteristics of the recipients, ranging from health status to genetic background can also be assumed to affect the impact of FMT. Due to the low number of studies conducted so far, there are many aspects that have not been answered yet: should the donors be of a similar age as the recipients?; should administration routes be oral or by enema?; at what age should FMT be applied?; how much material should be transplanted?; and what is the optimum number of transplantations?.

As discussed above, the literature on FMT in humans is extensive. However, while FMT in humans has mainly been used so far to treat diseased patients, in the case of most of the studies presented here dealing with pig production, FMT was practiced in non-diseased animals in an attempt to improve their health and/or feed efficiency.

As for most aspects of microbial ecology of the gut, the function profile more than the microbiota profile is relevant for the impact on the host. Accordingly, as discussed by Arrieta et al. [117], it is possible that the changes in phenotype in the recipients are not caused by the compositional characteristics of the engrafted microbiome, but by the ‘engrafted functions’, that is, bacterial-derived metabolites or components known to have strong metabolic and immunomodulatory effects. This is in line with the data of studies on CDI in humans by Weingarden et al. [118], Staley et al. [119], Smillie et al. [120], and Staley et al. [121], suggesting that complete engraftment is not necessary to resolve CDI, rather, that bacteria with certain functions potentially provide resistance to infection. Therefore, a combined composition-function analysis of the transplanted microbiome will provide better insight into the relevant mechanisms involved in the phenotypic change in recipient animals [117].

The level of engraftment necessary in pigs to improve feed efficiency or health/reduce risk of disease is not known. It could be hypothesized that, as for humans, establishment of a certain consortium of bacterial species/strains with the specific functions needed to improve these phenotypes in the recipients would be enough. However, the reason for practicing FMT is precisely that this beneficial consortium has not been identified. An example of a study trying to identify the specific bacteria or group of bacteria conferring the beneficial effects of FMT is that of Hu et al. [51] who, by performing various follow-up studies, showed that two specific bacterial species and via production of a specific bacteriocin could explain the reducing effect of FMT on diarrhea development in piglets.

Interestingly, not all the pig studies described report the level of engraftment, but rather differences among recipient groups. According to Arrieta et al. [117] this is not only the case for pig studies, but also many studies with HMA-mice do not report whether the transplanted microbiota has been established or only report changes at higher taxonomic levels.

While increased microbial diversity after FMT is considered an important contributor to the beneficial impact of FMT to CDI patients [76, 122], it should be remembered that these patients, besides having lower diversity due to CDI, contrary to piglets, have gone through an intensive antibiotic treatment, which is known to reduce microbial diversity [123], before FMT.

In order to achieve a successful establishment of the transplanted microbiota it could be argued that using donors of the same age as the recipients might be more appropriate. This is based on the hypothesis that animals of the same age have a more similar microbial ecosystem than those of different ages and therefore, the microbiota would more easily establish. On the other hand, using adult pigs as donors to much younger recipients could be based on the fact that during birth, piglets are colonised with the sow’s microbiota, which is considered beneficial for the general development of the offspring as compared to piglets born by cesarean-section and, therefore, without contact to the mother’s microbiota [124, 125]. The selection of the most appropriate donor, as mentioned above, needs to be further investigated.

The few studies published so far related to FMT in pigs as a strategy to improve health and/or performance do not allow a definitive conclusion on its effectiveness. Much more data is required in which the various factors that may affect the outcome are investigated.

Moreover, one area that, in our opinion, would also be worth covering to a greater extent is the use of FMT to treat certain pig diseases, for example, post-weaning diarrhoea. The microbiota of pigs suffering from diarrhea is dysbiotic, and FMT could help establishing a ‘normal’ population (which partially resembles the situation of CDI in humans).

If the results of using FMT as a strategy to improve feed efficiency or health in non-diseased pigs or to treat diseased pigs shows promise, putting this strategy into practice should be feasible. This would, for example, require treating the animals individually. While this initially may seem unviable from an economic point of view, it could be combined with routines in pig production currently requiring such effort, for example, iron supplementation and castration, or the individual treatment of pigs in quarantine or hospital pens.

On the other hand, in Critical Views in Gastroenterology and Hepatology [126], it was stated that ‘If we are still doing faecal transplants in 5 years’ time, we have failed. We will probably move into a situation in which a patient would be given a specific cocktail of organisms in a highly quality-controlled context’. There is no doubt that identifying the specific group of bacteria that lead to the searched outcome instead of using the whole faecal sample, with the risks and variability that can bring with it, would be preferable and should be searched for [51, 56, 121, 127]. In fact, studies using FMT can help acquiring this knowledge, just as it is the case in human research [118–121].

### Faecal microbiota transplantation in research

Faecal microbiota transplantation is also practiced when the pig is used as a model for humans, either by transplanting faeces from pigs to pigs [45, 46], or transplanting microbiota from humans to pigs to obtain HMA-pigs with the aim of investigating aspects relevant for humans [38–44, 89, 128, 129].

#### Description of studies

In order to test the hypothesis that the transplantation of gut microbiota could transfer certain immunological characteristics from donors to recipients, Xiao et al. [45] transplanted faecal microbiota of two pig lines, Yorkshire and Tibetan (Yorkshire being more susceptible to disease and Tibetan being more resistant), to commercial hybrid newborn piglets, followed by induction of acute colitis using dextran sulphate sodium (DSS).

Differences in microbiota composition and several immunological parameters, including lower levels of pro-inflammatory cytokines in Tibetan pigs, between the two donor breeds were observed. Oral DSS administration induced observable acute colitis only in the pigs colonized with the “Yorkshire microbiota”. Further, several inflammatory markers and expression of various molecules related to immune activation were promoted by DSS administration only in pigs that received the “Yorkshire microbiota”. Therefore, the transplantation with microbiota from Tibetan microbiota seemed to confer resistance to the inflammation induced by DSS, with less severe colonic haemorrhage and milder histological impact compared to FMT from Yorkshire donors. Lower abundance of Bacteroidetes and *Prevotella*, and higher abundance of *Fibrobacter* and *Lactobacillus* in the faeces of Tibetan pigs compared to Yorkshire pigs was speculated to be involved in the higher resistance to disease by Tibetan pigs. This is however, not proven.

Brunse et al. [46] used preterm piglets as a model to investigate the impact of FMT provided by different routes, i.e., either orally and rectally, or only rectally, on various parameters in relation to necrotizing enterocolitis (NEC) in preterm infants. This was done in an attempt to investigate the potential of FMT to prevent/treat NEC. Colon content from healthy suckling piglets was used as transplantation material. Five-day (the length of this study) survival was significantly reduced in the group administered oral + rectal FMT compared with controls (no FMT). Faecal microbiota-transplanted animals surviving until day 5 showed reduced growth compared with the control, but 60% relative reduction in NEC incidence. The results obtained in animals receiving FMT only via the rectum were more positive. In contrast to the results from piglets receiving oral + rectal FMT, 5-day survival was unaffected. Further, neither of the clinical parameters, motor activity, and growth rate were affected by rectal FMT; and importantly, FMT reduced the relative NEC incidence by 75% when administered only rectally.

In conclusion, oral FMT increased the risk of lethal sepsis, whereas rectal FMT protected against NEC without causing adverse effects. This indicated that the route of administration of FMT can be crucial for the output obtained. The authors proposed the introduction of lactate-utilizing bacteria such as *Bacteroides* as the mode of action behind the positive impact of FMT on NEC. This would change microbial metabolism towards lactate consumption and short chain fatty acids (SCFA) production. The combination of lower lactate and potential epithelial exchange of SCFA and bicarbonate would ensure a neutral luminal pH, which sustains a physical barrier and thereby protects the epithelial surface from bacterial invasion, and prevent mucosal damage and NEC.

As mentioned above, a different area of research of FMT in pigs is that in which pigs are used as a model for human research by obtaining HMA-pigs. That is, faeces from humans are transplanted to pigs with the purpose of obtaining pigs with human-like microbiota. This then allows studies to explore aspects of relevance for human gut ecology, nutrition, disease pathology, immunology, and drug discovery in pigs.

Conventional pigs, germ-free pigs, and germ-free pigs colonized with single or multiple bacterial strains have been used as model for humans since at least 1971 (e.g., [12, 130–136]. The pig- and human microbiome are more similar than, for example, the mouse and human microbiome [8], leading researchers to conclude that pig models are more appropriate as a surrogate for the human microbiome. However, the pig and human microbiomes are sufficiently different [8, 38, 137, 138], which, combined with the belief that the effects of single or multiple bacteria on the host do not reflect completely those of complex microbiota [110, 135, 137], has resulted in the need for better models. The premise of the HMA-pig model is that these animals respond to the experimental treatments in a similar way to humans, and more similarly than the mentioned conventional or single/multiple associated germ-free pigs. Further, the use of the pig rather than rodents, which have widely been used, including rodents with human-associated microbiota [117, 139–141], as a model for humans makes research more expensive, requires larger facilities, and often takes longer. The efforts made to establish a pig model are based on the belief that it is a better model than rodents due to the closer resemblance between pigs and humans with respect to the anatomy, physiology, immune system, metabolism, gut microbiome, and omnivorous diet [8, 136, 142–144].

Pang et al. [38] transplanted faeces from a boy to germ-free piglets delivered by cesarean section. According to the authors, they succeeded in establishing a donor-like microbial community, with minimal individual variation. Further, the microbial succession with aging of the ex-germfree piglets was also reported to be similar to that observed in humans. The latter was based on results showing that introduction of solid food to the piglets during the weaning period led to a reduction of bifidobacteria, which is in line with the shifts observed from breast-fed infants to adulthood in humans. Clustering analysis based on the fingerprints of Enterobacterial repetitive intergenic consensus sequence-PCR (ERIC-PCR) of intestinal microbiota of 10 unrelated healthy human individuals, five conventional piglets and two HMA-piglets showed that the human and HMA-piglet samples clustered together and the conventional piglet samples clustered in another group, which indicated that the DNA fingerprints of HFA piglets were more similar to that of humans than to the CV piglets.

Zhang et al. [40] had the aim of developing animal models enabling the manipulation of human microbiomes and the study of the impact of such perturbations on the host. They conducted three trials with a small number of animals (from 2 to 4 piglets) delivered by cesarian section, kept in sterile isolators, and fed with human infant milk formula or a sterile grower diet. Faeces from human adults or a breast-fed baby were transplanted to the piglets. The results obtained were variable, and according to the authors, microbiota composition of HMA-piglets transplanted with the infant donor tended to converge towards that of the donor, while that of HMA-piglets harboring the adult human microbiota did not. The few number of animals included and short sampling period makes it difficult to draw conclusions from these studies, though.

Zhang et al. [41] used the HMA-pig model to study aspects of rotavirus infection in humans and its relation to the gut microbiota. More specifically, the effect of a probiotic on the composition of the transplanted microbiota following rotavirus vaccination and challenge. The model was hypothesized to help testing interventions to prevent or treat infantile diarrhea caused by rotavirus and improve enteric health and immunity. Faeces from a cesarian section-delivered infant were transplanted to germ-free newborn piglets. Observing the bacterial composition of the human faeces and piglet colon digesta obtained by 16S rRNA gene amplicon sequencing, Zhang et al. [41] concluded that the recipient pigs carried microbiota similar to the human donor’s microbiota. However, this conclusion was not based on statistical analysis, what makes this conclusion uncertain.

Wen et al. [42] used HMA-pigs with the aim of establishing a model to study aspects of rotavirus infection in infants, too. Faeces from an infant were orally transplanted to germ-free piglets. They concluded that the HMA germ-free pig model is an appropriate model for studying immune responses to vaccines for human rotavirus infection and for evaluation of the immunomodulatory effects of probiotics. No analysis of the microbiota composition of the donor or recipient subjects was conducted, so it is unknown whether the donor microbiota established, or whether a similar output would have been seen if the animals had been transplanted with pig faeces. Furthermore, approximately 21% of the pigs became ill within several days after the oral administration of the microbiota from the infant donor, *Klebsiella oxytoca* being the likely cause of the illness. In line with these results, Wei et al. [39] reported that 17 of 24 piglets died due to the opportunistic pathogen *Klebsiella pneumoniae* present in the transplanted animals, which originated from an apparently healthy human donor of 11 years of age.

A series of studies, [44, 89, 128, 129] investigated various aspects of protein malnutrition and rotavirus infection in HMA-pigs. Kumar et al. [89] used the HMA model to test the hypothesis that malnutrition exacerbates rotavirus disease severity in infants. Faeces from a baby were transplanted to caesarean-delivered germ-free piglets. Seven days later, the microbiota of faeces and GI-tract of the piglets was analyzed and compared to the human microbiota transplanted gut. Despite differences in the microbial population between the donor and recipient both at phylum (e.g., Actinobacteria had a relative abundance of 40% in the donor and only ca. 2% in the recipients) and at lower taxonomic levels, the results were interpreted as showing a representative colonization of the pig intestines by the transplanted microbiota. This was based on the observation that the recipient pigs shared the majority of OTUs identified in the donor faeces but at different proportions. One of the differences between the transplanted microbiota and that found in the pigs was related to bifidobacterium (which is within the Actinobacteria phylum) being present in higher abundance in the transplanted microbiota than in that of the recipient piglets, which the authors attributed to the effect of the diet. The donor infant was breast-fed, which is known to promote bifidobacteria, whereas the piglets received formula. The authors also concluded that the results indicated that HMA-piglets on a malnourished diet displayed clinical symptoms mimicking the symptoms in malnourished infants. Similarly, Fisher et al. [44], Kumar et al. [89], Vlasova et al. [128], and Miyazaki et al. [129] evaluated this pig model to be a valid tool to investigate this research area. Microbiota composition of the donors or recipients was not reported in these studies, though.

Further, the HMA-pig model has been used with varying purposes. Shen et al. [142] studied the impact of short-chain fructo-oligosaccharides on the gut bacterial populations; Che et al. [145] investigated the effects of gut microbes from a different donor species on the intestinal morphology and mucosal immunity by comparing HMA-pigs with pig microbiota associated pigs.

#### Discussion of the results

The described studies of Xiao et al. [45] and Brunse et al. [46] showed the potential of using FMT to investigate the pathology of human diseases or as a mean of treating them. The studies revealed that factors including route of administration and donor have a marked impact on the evolution of the diseases investigated, and indicate that this pig model can help establishing the appropriate conditions to be applied for a successful FMT treatment before being tested in humans. Although by far proven, it could be speculated that a key factor relating to route of administration may be the compartment of the gut that is most affected by disease. With administration of FMT directly to the site of insult (for example the large intestine) being as important as the transplant material. However, it is also evident from the results that much knowledge is to be acquired before FMT can be used to prevent or treat these diseases.

Regarding the HMA-pig model, maximum engraftment would be considered the best outcome. The presence of bifidobacterium in HMA-pigs is considered by several authors as an important parameter when evaluating this model as superior to rodents to investigate aspects with relevance to the human microbiome [38, 137, 145]. This genus is considered an important member of the human gut, especially of breast-fed infants, and while some studies have shown that bifidobacteria do not readily colonize the rodent gut [146], other trials have shown establishment of bifidobacteria in mice [147]. However, a more detailed taxonomic analysis of the donor and recipient microbiota would be needed to conclude on the level of engraftment in the various animal species. In our opinion, this aspect has not been covered sufficiently, that is, comparison between the donor and recipient microbiota comunnity is often not statistically compared, which makes evaluation of the model difficult.

At the same time, it should be pointed out that, as previously discussed, the functional profile more than the microbiota profile is relevant for the impact on the host. Therefore, the arguments presented above suggesting that the changes in phenotype in the recipients are not caused by the compositional characteristics of the engrafted microbiome, but by the ‘engrafted functions [117], would be applicable here too.

All the studies described above using the HMA-pigs model presented worked with germ-free piglets at birth/delivered with cesarean-section and kept in isolators. These conditions make this type of study cumbersome and expensive. If pigs could be kept in non-sterilized conditions, the use of this model would most probably be more extended (cheaper, less specialized facilities needed, etc.), and longer-term studies would also be more affordable. This would require that the human microbiota established would remain even when the environment is not sterile. Diet is a crucial factor that impacts gut microbiota composition of pigs [16, 30, 148, 149]. Therefore, one possible model to try to maintain the transplanted microbiota for a longer time even in a non-sterilized environment (but also in germ-free models) could be by feeding the HMA-pigs a human-like diet during the study. To our knowledge, this has only been done in studies using the piglet as a model for infants, in which piglets were fed infant formula [46].

The importance of diet in the recipient animals in order to maintain the established transplanted microbiota is also discussed by Arrieta et al. [117] regarding mice, but there is no reason to think that this would not hold for pigs, too. Arrieta et al. [117] stated that it is likely that non-adapted bacterial strains are unable to utilize host-specific growth substrates (ie., glycans), and that reduced microbial adherence to the mucosal surface may lead to the loss of non-host adapted microbial species. Therefore, it is likely that a human microbiome that colonizes a mouse is more reliant on dietary growth substrates, as it has both limited ability to utilize host-derived growth substrates and limited interactions with surfaces that might support persistence even if growth rates are reduced.

## Conclusions and perspectives

With respect to the use of FMT in pigs when using this animal as a model for humans, Kirk [150], in a review on the use of larger animals and models in the context of organ transplantation, stated that animal models cannot be assumed to predict all aspects of subsequent human studies, but they can give data to meld with prior human experience. This encourages to continue pursuing improvement and development of pig (and other animals) as a model for humans. Data indicate though, that the pig has more similarities with the human regarding the GI-tract ecosystem than other non-primate species, what advocates for the inclusion of pig models at some stage during the process of investigating aspects related to humans within this area.

The use of FMT as a strategy to improve health, prevent or treat disease, or feed efficiency in pigs is in its infancy. The data available is scarce and does not allow strong conclusions to be drawn on the effectiveness of this strategy. The studies conducted so far have not reported consistent results, that is, some have shown improvements in the phenotype of the recipients whereas others have reported a negative impact.

**Fig. 1:**
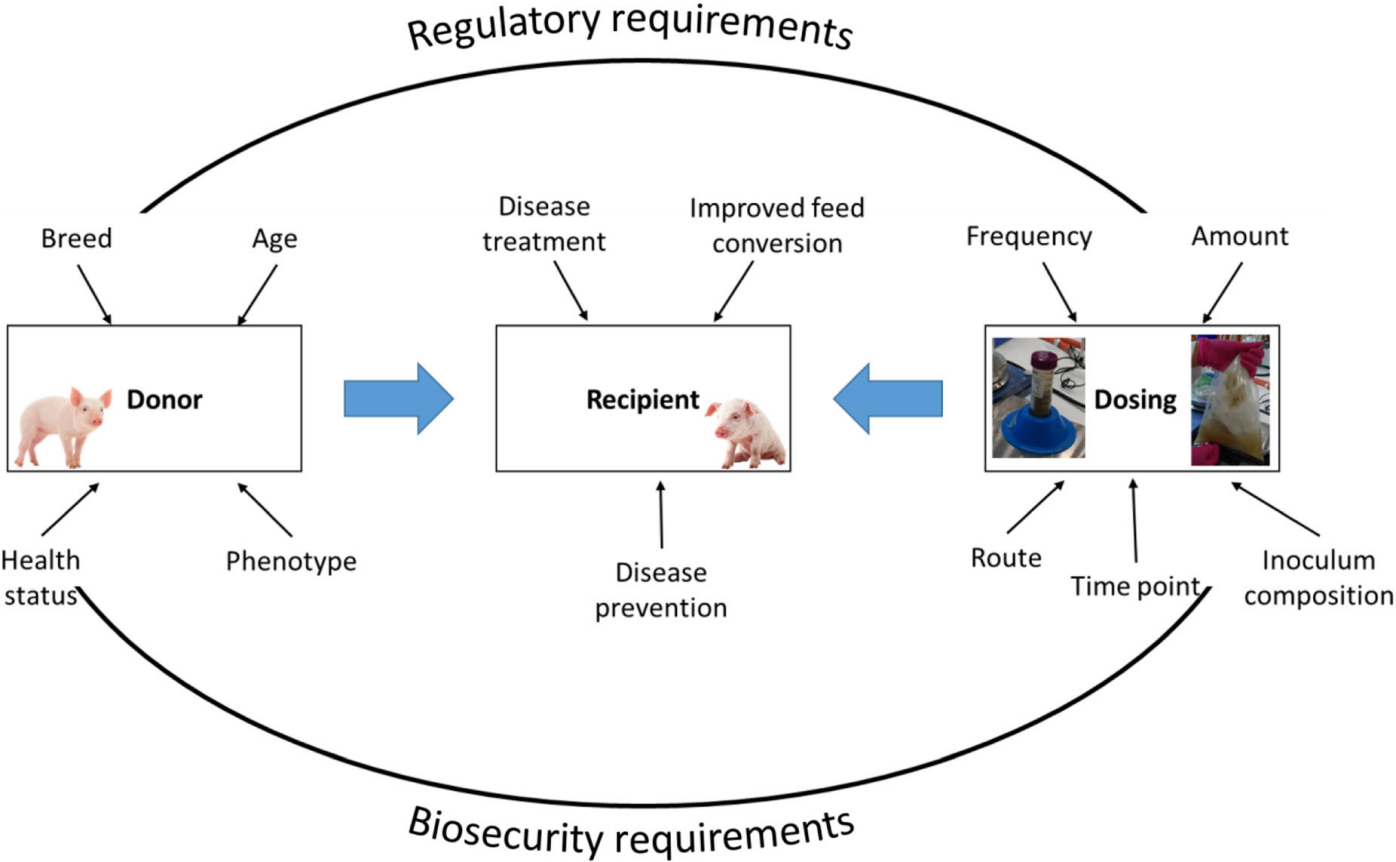
Factors to be considered when performing faecal microbiota transplantation in pigs

There is a high number of factors that may be crucial in order to obtain a successful outcome but have not been identified yet. Examples of these are: donor selection and screening, e.g., microbiota profile, presence of pathogenic microorganisms, age of donor with respect to recipient; time of transplantation, e.g., at birth, at weaning; frequency of transplantation; amount of transplant; factors affecting engraftment extent, e.g., as for organ transplantation, a match between donor and recipient might be important; and preparation of the transplant, e.g., only the pellet following centrifugation of the faecal material or the whole material, procedure to maximize viability. Another aspect is whether faecal/colon material is the most appropriate material to be transplanted regardless of the aim of the study, e.g., if the purpose is to improve health related to processes occurring in the small intestine, it would be prudent to assess whether transplanting small intestinal digesta might be more appropriate than faeces. An outline of important factors to be considered when practicing FMT are illustrated in Fig. 1.

Faecal microbiota transplantation is a strategy that results in resolution rates of around 90% for rCDI in humans, and, as such, it is worth investigating and developing FMT further in pig production both for production improvement and disease treatment aspects. On the other hand, it seems also clear that transplanting faecal material from an individual to another poses a risk of transmitting diseases or impairing other outcomes. Although not examined in this review, and acting as something of a caveat for FMT in large-scale intensive pig production, is the issue of biosecurity. As mentioned above, and identified for human medicine, screening of donor stock for pathogens is imperative. Multiple bacterial, parasitic, and viral agents can be transmitted via the faecal-oral route, and stock which have been treated with antimicrobials may harbor resistant bacteria which can be transferred to recipients, resulting in establishment of the resistant population, or transfer of resistance genes to the recipient’s commensal microbiota [151]. Additionally, it is common biosecurity practice to only allow stock onto a production unit via an integrated system, so a ‘one size fits all’ approach, where a large, easily accessible stock of FMT inoculum can be distributed to multiple producers is unlikely to be viable. It should be noted that the use of FMT in pigs would also be subjected to varying levels of regulations (based on country) for its use in pigs due to biosecurity/regulatory implications [152]. The regulatory barriers for the routine use of FMT in pigs require careful consideration and comprehensive risk-benefit assessment is also required to any prior routine use.

When more experience has been gained on FMT in pigs and taking advantage of this new knowledge, the future of the field, as proposed for humans, should be moving beyond faecal transplants and instead aiming to identify the organisms that are essential for a particular outcome, and then providing those organisms in a much simpler fashion than FMT. This would provide a safer and more sustainable alternative to faecal transplants. Probiotic products are in common use in human therapeutics, and to a lesser extent in animals, to alter GI-tract microbiota by providing specific microorganisms, however for the most part, they do not seem to colonize and persist in the gut [153–155]. Faecal microbiota transplantation consists of supplementing with an already established and competitive community, where much research will be needed to find specific strains that will work synergistically to provide the desired outcome, and be competitive enough to establish in the host.

According to Smillie et al. [120], whether this next generation of microbiome-based therapeutics effectively replaces FMT will depend on i) whether the “active ingredients” of FMT that carry out a desired mechanism can be identified, ii) whether these strains engraft in a patient’s gut, and iii) whether they are sufficiently abundant to produce a desired response. It could also be added, particularly in a field of large scale as intensive swine production, that a cost-effective, reproducible, quality controlled product would also be needed.

## Abbreviations

ANG-4: Angiogenin-4
*C. difficile*: *Clostridium difficile*
DMT1: Divalent metal transporter-1
DSS: Dextran sulphate sodium
*E. coli*: *Escherichia coli*
FMT: Faecal microbiota transplantation
GI-tract: Gastrointestinal tract
GLP-2: Glucagon-like peptide-2
HMA-pigs: Human microbiota-associated- pigs
IGF-1R: Insulin like growth factor-1 receptor
IL: Interleukin
LPS: Lipopolysaccharide
MUC1: Mucin 1
MUC2: Mucin 2
NEC: Necrotizing enterocolitis
OCLN: Occludin
OTU: Operational taxonomic unit
PCV-2: Porcine circovirus type 2
PCVAD: Porcine circovirus associated disease
PRRSV: Porcine reproductive and respiratory syndrome virus
rCDIs: Recurrent *Clostridium difficile* infections
SCFA: Short chain fatty acids
SLC_7_A_1_: Solute carrier family 7
SOD: Superoxide dismutase activity
ZNT-1: Zinc transporters-1
ZO-1: Zonula occludens-1
